# Circulating Omentin-1 Levels in Iron-Deficiency Anaemia and β-Thalassemia Trait: Associations with Iron and Metabolic Parameters

**DOI:** 10.3390/diagnostics16152419

**Published:** 2026-07-31

**Authors:** Kadriye Akpınar, Alev Lazoğlu Özkaya

**Affiliations:** 1 Department of Medical Biochemistry, School of Medicine, Burdur Mehmet Akif Ersoy University, Burdur 15200, Türkiye; 2 Occupational Health Unit, Burdur Provincial Health Directorate, Burdur 15040, Türkiye; 3 Department of Biochemistry, Erzurum Regional Training and Research Hospital, Erzurum 25050, Türkiye; alev.lazogluozkaya@saglik.gov.tr

**Keywords:** omentin-1, intelectin-1, iron-deficiency anaemia, β-thalassemia trait, iron metabolism, Hba1c

## Abstract

**Background/Objectives:** Omentin-1 (intelectin-1) is an adipocytokine with anti-inflammatory, insulin-sensitizing, and iron-regulatory properties. This study aimed to compare circulating omentin-1 levels in patients with (IDA) and β-thalassemia trait and to evaluate their associations with haematological, iron-related, and metabolic parameters. **Methods:** A total of 90 adults (*n* = 90), including 29 patients with IDA, 31 individuals with β-thalassemia trait, and 30 healthy controls. Haematological indices, iron parameters, biochemical markers, and plasma omentin-1 levels were measured. Group comparisons, correlation analyses, multivariable linear regression, and receiver operating characteristic (ROC) curve analyses were performed. **Results:** Plasma omentin-1 concentrations were significantly lower in patients with hypochromic microcytic anaemia than in healthy controls (36.4 vs. 147.7 ng/mL, *p* < 0.001). Among subgroups, the lowest levels were observed in IDA patients, followed by β-thalassemia trait and controls (*p* < 0.001). Omentin-1 showed positive correlations with haemoglobin, MCV, MCH, ferritin, serum iron, total cholesterol, HDL-C, and LDL-C, whereas negative correlations were observed with age, HbA1c, glucose, RDW, and total iron-binding capacity (all *p* < 0.05). In multivariable analysis, age (β = −0.215, *p* = 0.046), female sex (β = 0.305, *p* = 0.028), and MCV (β = 0.334, *p* = 0.015) were independent predictors of omentin-1 levels. ROC analysis demonstrated excellent discrimination between IDA and controls (AUC = 0.93) and good discrimination between β-thalassemia trait and controls (AUC = 0.79). **Conclusions:** Omentin-1 levels are reduced in IDA and β-thalassemia trait and are closely associated with iron status, erythroid indices, and metabolic parameters. Omentin-1 may serve as an adjunct biomarker for hypochromic microcytic anaemia, particularly in distinguishing affected individuals from healthy subjects. However, its limited utility in distinguishing iron-deficiency anaemia from β-thalassemia trait indicates that it should be used alongside established haematological and iron-related parameters.

## 1. Introduction

Anaemia is a condition characterized by a reduction in red blood cell mass, leading to the inability of circulating red blood cells to meet the physiological oxygen-carrying needs of the body, and is detected when haemoglobin (Hb), haematocrit (Hct), and red blood cell counts (RBCs) fall below the accepted normal values for a given age and sex. According to the World Health Organization (WHO) guideline on haemoglobin cut-offs, anaemia is defined as a haemoglobin concentration below 13.0 g/dL in adult men, below 12.0 g/dL in non-pregnant adult women, and below 11.0 g/dL in pregnant adult women. The guideline recommends adjusting haemoglobin concentrations for altitude and smoking status, when applicable, whereas no adjustments are recommended for inflammation, infection, or genetic ancestry/ethnicity [1]. The causes of anaemia include nutritional deficiencies, genetic haemoglobin disorders, chronic inflammatory diseases, chronic kidney disease, bone marrow disorders, infectious conditions and various conditions leading to blood loss or increased red blood cell destruction [2]. Anaemia is a common and significant global health problem, and the most frequent type is hypochromic microcytic anaemia. Iron-deficiency anaemia (IDA) (60%) and β-thalassemia syndromes (5%) are among the main causes of hypochromic microcytic anaemia [3,4]. β-thalassemia carrier (thalassemia minor) status is the most common clinical form among thalassemia syndromes, with an estimated global prevalence of approximately 1–1.5%. β-thalassemia is an autosomal recessive hemoglobinopathy caused by mutations in the HBB gene that reduce or abolish β-globin synthesis. The resulting globin chain imbalance causes ineffective erythropoiesis and chronic haemolytic anaemia, with clinical manifestations ranging from asymptomatic carrier states to transfusion-dependent disease [1,4]. In the diagnosis of both conditions, laboratory findings from the complete blood count, such as RBC (red blood cell count), Hb (haemoglobin), MCV (mean corpuscular volume), MCH (mean corpuscular haemoglobin), MCHC (mean corpuscular haemoglobin concentration), and RDW (red cell distribution width), are used. In identifying β-thalassemia carriers, in addition to haematological methods, quantification of HbA2 and HbF is performed, and when necessary, molecular methods are used as advanced investigations [5]. The earliest measurable laboratory finding of IDA is a decrease in ferritin levels. Subsequently, serum ferritin, iron levels and transferrin saturation decrease; total iron-binding capacity (TIBC) increases; and finally, Hb levels decline [6,7]. Accurate differentiation between IDA and β-thalassemia trait is essential, as carriers may be inappropriately treated with iron supplementation when microcytosis is mistaken for iron deficiency. Such treatment is ineffective in the absence of iron deficiency and may lead to excessive iron accumulation with prolonged use, whereas iron therapy is appropriate when iron deficiency coexists with β-thalassemia trait [8,9].

Omentin-1 is also known as intelectin-1, intestinal lactoferrin receptor, endothelial lectin HL-1, or galactofuranose-binding lectin. It is a novel adipocytokine with anti-inflammatory, antioxidant, anti-apoptotic, and antimicrobial effects. In addition, omentin-1 has been implicated in immune regulation and host defence mechanisms [10,11]. It is expressed in various cell types, including endothelial cells, mesothelial cells, smooth muscle cells, airway and intestinal goblet cells, and vascular cells [12]. Particularly in metabolic diseases, circulating omentin-1 has excellent potential as a non-invasive biomarker. Omentin-1 also plays an important role in physiological iron regulation. It is highly expressed in the brush border of the small intestinal epithelium, and its main function is to facilitate the uptake of iron-bound lactoferrin by enterocytes via receptor-mediated endocytosis and its subsequent intracellular transport [13]. As a lactoferrin receptor, omentin-1 has been shown to facilitate lactoferrin-mediated iron uptake, particularly in the developing intestine of infants. Although its role in intestinal iron absorption in adults remains uncertain, omentin-1 may contribute to systemic iron homeostasis through lactoferrin-related pathways. Given that conventional biomarkers used to assess iron status, including transferrin saturation and ferritin, can be influenced by inflammation, malnutrition, and chronic disease, thereby limiting their diagnostic accuracy in certain clinical settings, investigation of novel biomarkers involved in iron metabolism may provide additional diagnostic value [14]. Therefore, molecules involved in iron metabolism, such as omentin-1, may provide additional diagnostic value as potential biomarkers of iron deficiency. Nevertheless, it should be acknowledged that the clinical application of omentin-1 remains limited by the lack of standardized assays and reference ranges, limited assay availability, and relatively high analytical costs.

Although omentin-1 has been implicated in iron metabolism, its diagnostic value in differentiating iron-deficiency anaemia (IDA) from β-thalassemia trait remains unclear. To our knowledge, no previous study has directly compared circulating omentin-1 levels between patients with IDA and β-thalassemia trait. Therefore, we aimed to compare circulating omentin-1 levels and their associations with iron and metabolic parameters in patients with hypochromic microcytic anaemia due to iron deficiency or β-thalassemia trait, as well as in healthy controls, and to evaluate the potential diagnostic utility of omentin-1 in differentiating these two common causes of hypochromic microcytic anaemia.

## 2. Materials and Methods

### 2.1. Study Design and Population

A total of 90 individuals aged over 18 years (mean ± standard deviation: 32.12 ± 12.88; minimum–maximum: 18–69), including 46 males and 44 females, were enrolled in this study. Eligible cases consisted of consecutive participants who applied to the Occupational Health and Safety Centre for routine occupational health examinations as part of the annual screening program conducted by the Burdur Provincial Health Directorate in 2024. All participants were of Turkish nationality and the same residency (Burdur, Türkiye). We included participants with no missing data. Individuals with chronic diseases, active infections, regular medication use, and anaemia other than iron-deficiency anaemia or β-thalassemia trait were excluded from the study. The study population consisted of 30 healthy controls and 60 patients diagnosed with hypochromic microcytic anaemia. The patient group was composed of 29 patients with IDA and 31 individuals with β-thalassemia trait. IDA was diagnosed according to World Health Organization (WHO) criteria [1]. Iron-deficiency anaemia was diagnosed according to current international guidelines using haemoglobin (<13.0 g/dL in men and <12.0 g/dL in non-pregnant women), serum ferritin (<30 µg/L in the absence of inflammation), transferrin saturation (<20%), and red blood cell indices (MCV <80 fL, MCH <27 pg, and increased RDW) [3,15]. β-thalassemia trait was diagnosed according to international guidelines based on complete blood count findings consistent with microcytosis (typically an increased RBC count with MCV <80 fL and MCH <27 pg) together with elevated HbA2 levels (≥3.5%) determined by HPLC [16]. For all participants, interviews were carried out by the same physician to obtain information on demographic and medical history.

### 2.2. Procedures

Blood collection was performed under similar conditions early in the morning, after 12 h of fasting. Blood samples were collected from the antecubital vein after the participants had rested in a seated position. Samples for biochemical and hormonal analyses were collected into gel vacuum tubes (Vacusera, Türkiye), whereas whole blood samples for HbA1c, complete blood count, and omentin-1 analyses were collected into ethylenediaminetetraacetic acid (EDTA) tubes (Vacusera, Türkiye). For serum analyses, blood samples were allowed to clot at room temperature for 30 min and were then centrifuged at 1500× *g* for 10 min to separate the serum in the laboratory. Fasting glucose (FG), total cholesterol (TC), triglycerides (TGs), high-density lipoprotein cholesterol (HDL-C), and low-density lipoprotein cholesterol (LDL-C) levels were measured in serum using spectrophotometric methods on the Architect c8000 autoanalyzer (Abbott Laboratories, Abbott Park, IL, USA). LDL cholesterol levels were calculated using the Friedewald equation, which is applicable when lipid concentrations are expressed in mg/dL and triglyceride (TG) levels are below 400 mg/dL, according to the following formula: LDL = TC − [HDL + (TG/5)]. Ferritin levels were measured in serum using an immunoassay method on the Atellica IM autoanalyzer (Siemens Healthineers, Erlangen, Germany). HbA1c levels were analysed from whole blood using ion-exchange high-performance liquid chromatography (HPLC) in accordance with the National Glycohemoglobin Standardization Program guidelines, on the Lifotronic H9 analyser (Lifotronic Technology Co., Ltd., Shenzhen, China). Complete blood count parameters were measured from whole blood using the CELL-DYN Ruby fully automated haematology analyser (Abbott Laboratories, Abbott Park, IL, USA). For the diagnosis of β-thalassemia trait, haemoglobin fractions including HbA2, HbA0 and HbF peaks were analysed by ion-exchange high-performance liquid chromatography (HPLC) using the D-10™ Haemoglobin Testing System (Bio-Rad Laboratories, Hercules, CA, USA). For omentin-1 level measurement, whole blood samples were centrifuged at 1500× *g* for 10 min to separate the plasma. Plasma samples for omentin-1 analysis were aliquoted into Eppendorf tubes and stored at −20 °C for up to 6 months until batch analysis. After thawing, the samples were brought to room temperature and analysed using a commercially available sandwich enzyme-linked immunosorbent assay (ELISA) kit (Human Omentin-1 ELISA Kit, BT LAB, Shanghai, China; Cat. No. E0155Hu) according to the manufacturer’s instructions. Absorbance was measured at 450 nm using RT-2100C microplate reader (Rayto,Life and Analytical Sciences Co., Ltd., Shenzhen, China). The assay had a linear range of 2–600 ng/mL, sensitivity 1.03 ng/mL, an intra-assay coefficient of variation of <8%, and an inter-assay coefficient of variation of <10%. A reference range for omentin-1 was not available, as it was not provided by the manufacturer of the ELISA kit. Subsequently, biochemical parameters, complete blood count findings, and omentin-1 levels were compared among the groups.

### 2.3. Statistical Analysis

The sample size was determined based on a priori power analysis conducted using G*Power 3.1 software (Faul, Erdfelder, Lang and Buchner; Heinrich Heine University, Düsseldorf, Germany, 2020). Assuming a large effect size (Cohen’s f = 0.40), a significance level (α) of 0.05, and a statistical power of 80%, the planned sample size was considered sufficient to detect clinically meaningful between-group differences. The final study included 90 participants (29 patients with iron-deficiency anaemia, 31 individuals with β-thalassemia trait, and 30 healthy controls), indicating that the study was adequately powered. Statistical analyses were performed using SPSS version 27 for Windows (IBM Corp., Armonk, NY, USA). The normality of continuous variables was assessed using the Kolmogorov–Smirnov test. Normally distributed variables were expressed as mean ± standard deviation (SD), whereas non-normally distributed variables were presented as median (Q1–Q3) where Q1 (first quartile) and Q3 (third quartile) represent the 25th and 75th percentiles, respectively. A one-way analysis of variance (ANOVA) for normally distributed variables or the Kruskal–Wallis test for not normally distributed variables were used for between group comparisons in the cases of more than two groups of continuous variables. Significance values were adjusted by the Bonferroni correction for multiple tests. Effect sizes were calculated to assess the magnitude of differences between groups. For independent samples *t*-tests, Cohen’s d was used, representing the standardized mean difference between two groups [small (0.2–0.49), medium (0.5–0.79), large (≥0.8)]. For Mann–Whitney U tests, effect size was calculated using r = Z/√N [small (0.10–0.29), medium (0.30–0.49), and large (≥0.50)]. For Kruskal–Wallis tests, epsilon squared (ε^2^) was used to estimate the proportion of variance explained by group differences [small (0.01–0.05), medium (0.06–0.13), large (≥0.14)]. These effect size measures were selected according to the statistical test applied and the distribution characteristics of the variables.

Relationships between plasma omentin-1 levels and continuous variables were assessed using Spearman’s rank correlation analysis because plasma omentin-1 levels were not normally distributed. Correlation coefficients (r) were classified as follows: values between 0.00 and 0.25 indicated no or very weak correlation, 0.25–0.50 weak correlation, 0.50–0.75 moderate correlation, and 0.75–1.00 strong correlation. The normality of the regression residuals was assessed using histograms and normal probability (Q–Q) plots. Variables showing potential associations with plasma omentin-1 levels in univariable analyses (*p* < 0.20) were considered candidates for inclusion in the multivariable linear regression model. Candidate variables included age, HbA1c, haemoglobin, MCV, MCH, MCHC, RDW, ferritin, serum iron, TIBC, glucose, total cholesterol, HDL-C, and LDL-C. To avoid multicollinearity, clinically related variables were not included simultaneously in the final model. Interaction terms between independent variables were not evaluated because the primary objective of the analysis was to identify independent predictors, and the sample size was not considered sufficient to support reliable interaction analyses. A receiver operating characteristic (ROC) curve analysis and calculation of the area under the curve (AUC) were carried out to evaluate the ability of serum omentin-1 to correctly distinguish between patients with IDA and controls, patients with β-thalassemia trait and controls, and patients with IDA and β-thalassemia trait. The optimum diagnostic cut-off point for the examined population was chosen to maximize clinical sensitivity and specificity. The level of statistical significance was set at 0.05 for all tests performed. Figures were generated using GraphPad Prism version 10.0 (GraphPad Software, San Diego, CA, USA).

## 3. Results

A total of 90 participants were included in the study, comprising 60 patients with hypochromic microcytic anaemia and 30 healthy controls. Among the patient group, individuals with β-thalassemia trait and IDA were evaluated separately in subgroup analyses. The descriptive characteristics of the study population (*n* = 90), including age, haematological indices, iron parameters, and biochemical measurements, are presented in Table 1. Omentin-1 concentrations showed substantial inter-individual variability (range: 2.00–376.80 ng/mL); however, all measured values were within the analytical measurement range of the ELISA (2–600 ng/mL). The observed variability was considered to reflect biological differences between study groups rather than analytical imprecision. CRP levels were below the clinical cut-off value (<5 mg/L) in all participants. Given that the lower reporting limit of the assay was 2 mg/L, the limited variability observed in CRP values reflects the assay reporting characteristics rather than a data-related issue. These findings suggest no evidence of systemic inflammation in the study population.

Sex-based comparisons of demographic, haematological, iron-related, and biochemical parameters are presented in Table 2. Statistically significant differences were observed in age, omentin-1 levels, RBC, haemoglobin, Mentzer index, ferritin, iron, TIBC, glucose, total cholesterol, and HDL-C (*p* < 0.05), whereas the remaining parameters showed no significant differences between female and male participants.

**Figure 1:**
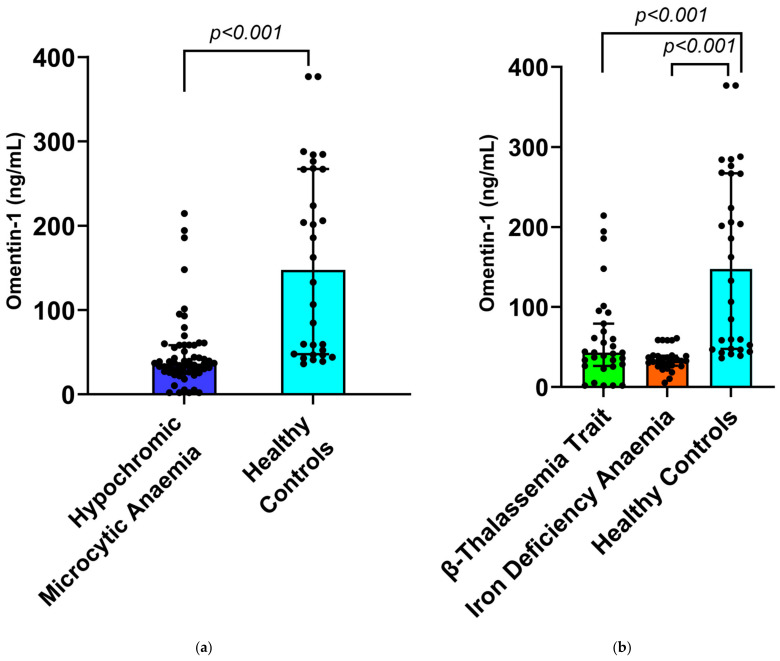
(**a**) Omentin-1 levels in patients with hypochromic microcytic anaemia and healthy controls. (**b**) Omentin-1 levels in patients with β-thalassemia trait, iron-deficiency anaemia, and healthy controls.

The clinical and biochemical characteristics of patients with hypochromic microcytic anaemia were compared with those of healthy controls, as shown in Table 3. The large effect sizes observed for haemoglobin and erythrocyte indices (MCV and MCH) further support the substantial haematological differences between patients with hypochromic microcytic anaemia and healthy controls. Statistically significant differences were identified in omentin-1 levels, HbF, HbA0, HbA2, red blood cell indices, Mentzer index, iron metabolism parameters, glucose, total cholesterol, and LDL cholesterol (*p* < 0.05). Figure 1a illustrates plasma omentin-1 levels in patients with hypochromic microcytic anaemia and healthy controls. In contrast, age, HbA1c, triglycerides, HDL cholesterol, and CRP did not differ significantly between the groups.

To further evaluate subgroup-specific differences, patients with hypochromic microcytic anaemia were classified into β-thalassemia trait and IDA groups and compared with healthy controls, as presented in Table 4. Statistically significant differences were observed in omentin-1 levels, HbF, HbA0, HbA2, red blood cell indices (RBC, Hb, MCV, MCH, MCHC, and RDW), Mentzer index, iron metabolism parameters (ferritin, iron, and TIBC), and total cholesterol (*p* < 0.05). Omentin-1 levels were significantly lower in the IDA group compared with both the β-thalassemia trait and healthy control groups, while β-thalassemia trait participants also had significantly lower levels than healthy controls (all post-hoc *p* < 0.05). Figure 1b illustrates plasma omentin-1 levels among the β-thalassemia trait, IDA, and healthy control groups. In contrast, age, HbA1c, glucose, triglycerides, HDL-C, LDL-C in the overall comparison, and CRP showed no significant differences between the groups.

The associations between plasma omentin-1 levels and haematological and biochemical parameters were evaluated using Spearman correlation analysis, as presented in Table 5. Significant correlations were identified between plasma omentin-1 levels and several haematological and biochemical variables. Omentin-1 was positively correlated with haemoglobin, erythrocyte indices, iron parameters, and lipid profile components (all *p* < 0.05), whereas it was negatively correlated with age, HbA1c, RDW, glucose, and TIBC (all *p* < 0.05). The inverse correlation between age and omentin-1 levels remained statistically significant after adjustment for sex (partial r = −0.259, *p* = 0.014), indicating that the association was not solely explained by sex differences. In the multivariable linear regression analysis, the overall model was statistically significant (F = 4.504, *p* < 0.001), explaining 27.8% of the variance in circulating omentin-1 levels (R^2^ = 0.278; adjusted R^2^ = 0.216). After multivariable adjustment, age (β = −0.215, *p* = 0.046), female sex (β = 0.305, *p* = 0.028), and MCV (β = 0.334, *p* = 0.015) were identified as independent predictors of circulating omentin-1 levels, whereas haemoglobin, ferritin, glucose, and HDL-C were not independently associated with omentin-1 concentrations. No significant multicollinearity was detected among the independent variables (tolerance range: 0.35–0.78; VIF range: 1.28–2.89).

**Figure 2:**
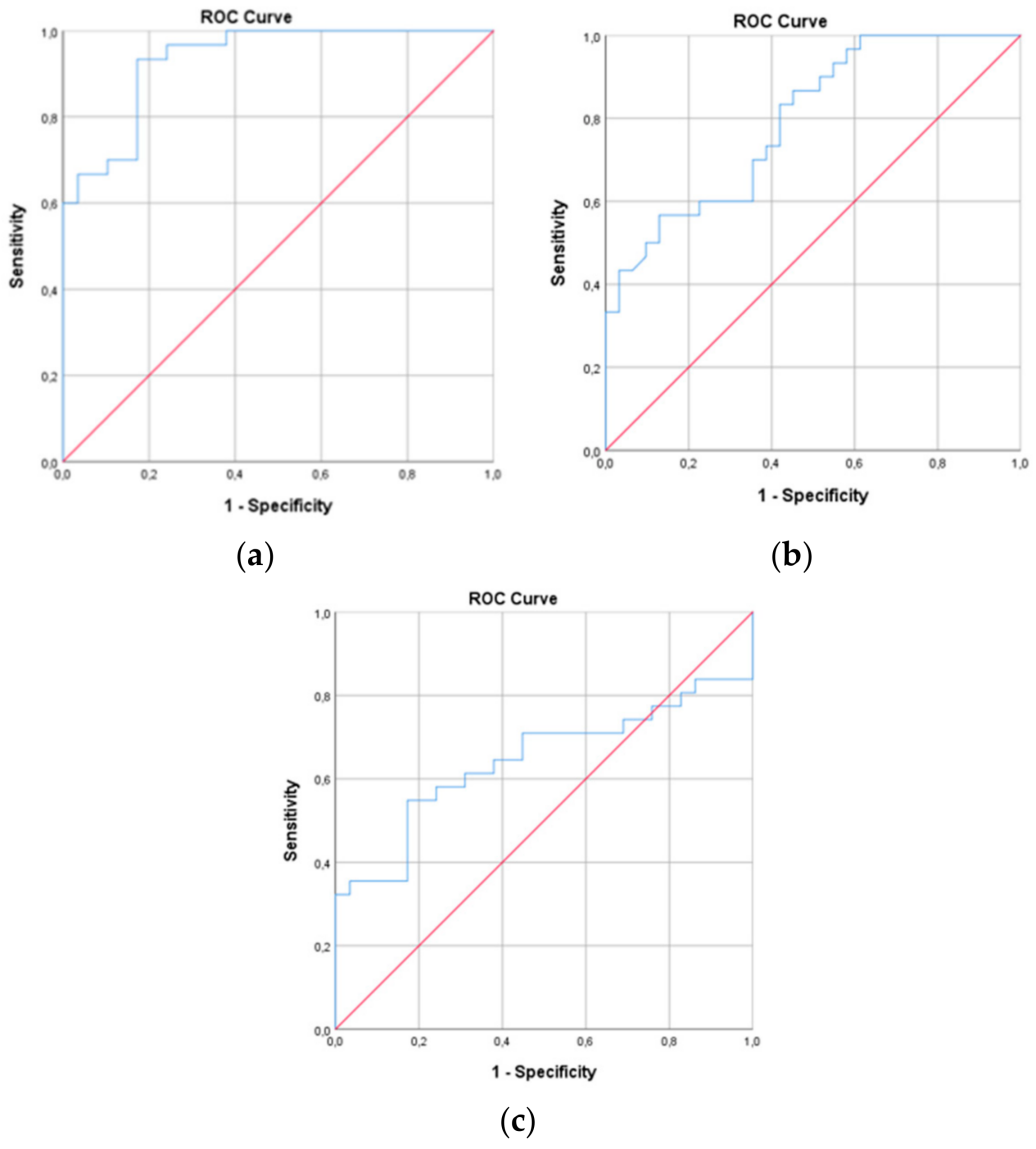
Receiver operating characteristic (ROC) curves of plasma omentin-1 levels for distinguishing iron-deficiency anaemia from healthy controls (AUC = 0.93, 95% CI: 0.87–0.99, *p* < 0.001) (**a**), β-thalassemia trait from healthy controls (AUC = 0.79, 95% CI: 0.68–0.90, *p* < 0.001) (**b**), and iron-deficiency anaemia from β-thalassemia trait (AUC = 0.64, 95% CI: 0.49–0.79, *p* = 0.055) (**c**).The blue line represents the ROC curve, and the red diagonal line represents the line of no discrimination (reference line; AUC = 0.50).

ROC curve analysis was performed to evaluate the diagnostic performance of plasma omentin-1 levels in differentiating IDA, β-thalassemia trait and healthy controls. Plasma omentin-1 demonstrated excellent discriminatory ability for distinguishing IDA from healthy controls (AUC = 0.93, 95% CI 0.87–0.99, *p* < 0.001) and good performance for differentiating β-thalassemia trait from healthy controls (AUC = 0.79, 95% CI 0.68–0.90, *p* < 0.001). In addition, omentin-1 did not significantly distinguish IDA from β-thalassemia trait (AUC = 0.64, 95% CI 0.49–0.79, *p* = 0.055), indicating limited diagnostic utility for differentiating these two conditions. The optimal cut-off value was 40.6 ng/mL, yielding a sensitivity of 54.8% and a specificity of 82.8% according to the Youden index (J = 0.376) for distinguishing IDA from β-thalassemia trait. The ROC curves for the three comparisons are presented in Figure 2.

## 4. Discussion

This study investigated circulating omentin-1 levels in patients with IDA, β-thalassemia trait, and healthy controls, and evaluated its associations with haematological, iron-related, and metabolic parameters. The main findings can be summarized as follows: plasma omentin-1 levels were significantly lower in hypochromic microcytic anaemia compared to healthy controls; both IDA and β-thalassemia trait were associated with reduced omentin-1 levels, with the lowest values observed in IDA; omentin-1 showed significant correlations with erythroid indices, iron status markers, and metabolic parameters; and although omentin-1 demonstrated good diagnostic performance in distinguishing anaemic patients from healthy individuals, its ability to differentiate IDA from β-thalassemia trait was limited.

The significantly reduced omentin-1 levels in patients with IDA suggest a potential link between iron metabolism and this adipocytokine. Omentin-1 is primarily known for its anti-inflammatory and insulin-sensitizing properties, but emerging evidence also indicates a role in iron homeostasis through its function as a lactoferrin receptor in the intestinal epithelium [12]. Experimental studies have shown that intelectin-1 facilitates lactoferrin-mediated iron uptake in enterocytes, thereby contributing to systemic iron absorption [13,17]. However, whether circulating omentin-1 concentrations reflect intestinal expression or activity remains unclear. Therefore, the lower circulating omentin-1 levels observed in IDA should be interpreted cautiously. They may be associated with alterations in iron metabolism, but the underlying biological mechanisms remain speculative. Furthermore, because of the cross-sectional design of the present study, it cannot be determined whether reduced circulating omentin-1 represents a cause, a consequence, or simply a correlate of iron deficiency. The significantly lower omentin-1 levels observed in IDA compared to β-thalassemia trait are particularly interesting. IDA is characterized by depleted iron stores, whereas β-thalassemia trait typically presents with ineffective erythropoiesis but relatively preserved or even increased iron stores due to increased absorption. Therefore, the more pronounced reduction in omentin-1 in IDA is consistent with a closer association between circulating omentin-1 and iron availability than with erythropoietic activity alone, although this interpretation requires confirmation in longitudinal and mechanistic studies. Bolignano et al. demonstrated a positive association between circulating omentin-1 and iron status markers, showing that omentin-1 levels decrease in states of iron depletion and increase following iron supplementation [14]. Although ferritin and serum iron levels were largely within normal ranges in our β-thalassemia trait, the significantly lower omentin-1 concentrations may reflect subtle alterations in iron homeostasis rather than absolute iron deficiency [18]. This finding suggests that circulating omentin-1 may be influenced not only by iron stores but also by erythropoietic activity and iron utilization. Chronic low-grade ineffective erythropoiesis, a hallmark of β-thalassemia trait, may increase erythroid iron demand and subtly disrupt iron homeostasis, thereby contributing to lower circulating omentin-1 levels. This interpretation is further supported by the findings of Guimarães et al. [18], who demonstrated that β-thalassemia carriers exhibit increased soluble transferrin receptor and erythropoietin levels despite relatively preserved iron stores, indicating persistent ineffective erythropoiesis and altered iron metabolism even in clinically asymptomatic carriers. An important finding of the present study is that circulating omentin-1 concentrations were significantly reduced in individuals with β-thalassemia trait, in contrast to the elevated levels previously reported in patients with clinically overt thalassemia by Alsaady et al. [19] and Al-Sammari et al. [20], who attributed these findings primarily to disease-related inflammatory and metabolic alterations. This discrepancy is likely explained by differences in the study populations. Whereas the previous studies included patients with clinically overt thalassemia, our study focused exclusively on individuals with β-thalassemia trait, who generally have milder disease, preserved iron stores, and no transfusion requirement. These clinical differences are likely associated with a lower inflammatory burden and distinct metabolic characteristics, which may partly explain the contrasting omentin-1 profiles observed across studies. In our study, β-thalassemia carriers showed lower omentin-1 concentrations despite normal CRP and ferritin levels and near-normal iron parameters. This finding suggests that inflammation is unlikely to be the primary determinant of omentin-1 in β-thalassemia trait. Instead, mild but chronic ineffective erythropoiesis, which persists even in carriers, may contribute to altered adipokine regulation and the observed reduction in circulating omentin-1 levels. The negative association between omentin-1 and RDW, as well as its positive relationship with MCV and MCH, suggests that lower omentin-1 levels are associated with more substantial microcytosis and anisocytosis. These findings support the hypothesis that omentin-1 may be indirectly linked to erythropoietic efficiency and iron utilization. Although direct evidence linking omentin-1 to red cell indices remains limited, previous studies have demonstrated significant associations between omentin-1 and iron-related parameters, supporting a potential role of this adipocytokine in iron homeostasis [14,21] although data in anaemic populations remain limited.

Regarding metabolic parameters, omentin-1 showed significant correlations with glucose and lipid parameters in this study. The inverse relationship with glucose and HbA1c is consistent with its insulin-sensitizing effects [22,23]. Fasting glucose was modestly but significantly higher in the overall hypochromic microcytic anaemia group than in healthy controls, although values remained within the normal range, which may be consistent with reduced insulin sensitivity accompanying lower circulating omentin-1 levels. Omentin-1 is predominantly secreted by visceral adipose tissue and is known to enhance insulin-stimulated glucose uptake in adipocytes. Therefore, lower omentin-1 levels in anaemic individuals may reflect broader metabolic alterations associated with chronic haematological stress rather than indicating a causal adaptive response. The observed associations with lipid parameters, particularly HDL-C, further support the role of omentin-1 in metabolic regulation. However, the positive correlations with total cholesterol and LDL-C were unexpected, given the anti-atherogenic and cardioprotective properties of omentin-1 and its generally inverse association with adverse lipid profiles reported in previous studies [24,25]. This discrepancy may be related to differences in study populations, as our study consisted of individuals with hypochromic microcytic anaemia rather than patients with obesity, diabetes, or established cardiovascular disease. Nevertheless, the directionality and clinical significance of these associations in anaemic populations remain uncertain and warrant further investigation.

The diagnostic performance analysis revealed that omentin-1 has excellent discriminatory ability in distinguishing patients with IDA from healthy controls (AUC = 0.93) and good performance for differentiating β-thalassemia trait from healthy controls (AUC = 0.79). The high AUC values suggest that omentin-1 may serve as a potential adjunct biomarker for identifying hypochromic microcytic anaemia. However, its performance in differentiating IDA from β-thalassemia trait was poor (AUC = 0.64) and not statistically significant. Furthermore, the cut-off value of 40.6 ng/mL for distinguishing IDA from β-thalassemia trait yields low sensitivity (54.8%) and moderate specificity (82.8%). Accordingly, omentin-1 cannot replace established diagnostic tools such as red blood cell indices, the Mentzer index, iron studies, and HbA2 quantification, which remain the cornerstone of differentiating IDA from β-thalassemia trait. This reinforces the conclusion that omentin-1 is not a useful discriminator between these two conditions. Instead, its potential clinical value may lie in complementing conventional laboratory markers by providing additional information on the metabolic and iron-related alterations associated with hypochromic microcytic anaemia, rather than serving as a standalone diagnostic marker. This limitation is clinically important, as one of the major diagnostic challenges in microcytic anaemia is the differentiation between these two conditions. The overlap in omentin-1 levels between IDA and β-thalassemia trait may be attributable to shared pathophysiological mechanisms, including ineffective erythropoiesis and altered iron handling, which may similarly influence omentin-1 expression [12,13,14].

The multivariable regression analysis suggested that age, female sex and MCV were independent predictors of omentin-1 levels, whereas ferritin, haemoglobin, glucose, and HDL-C were not independently associated in the final model. These results suggest that alterations in red blood cell indices, particularly MCV, may better reflect the haematological consequences of iron deficiency and erythropoietic alterations than ferritin alone. Consequently, ferritin was not independently associated with circulating omentin-1 after adjustment for the other variables in the model. Age-related declines in omentin-1 have also been reported previously and may reflect age-associated metabolic and inflammatory changes. Female sex also emerged as an independent predictor of circulating omentin-1 levels after multivariable adjustment, suggesting that sex-related biological differences contribute to omentin-1 variability independently of haematological and metabolic parameters.

Several limitations of this study should be acknowledged. First, the cross-sectional design precludes causal inference regarding the relationship between omentin-1 and iron metabolism. Second, participants were consecutively recruited from a single centre, which may have introduced selection bias and limited the generalizability of the findings. Moreover, all participants were recruited from a single province in Türkiye; therefore, the findings may not be generalizable to populations with different ethnic, genetic, environmental, or nutritional backgrounds. Third, the relatively modest sample size may have reduced the statistical power for subgroup analyses and further limited the generalizability of the results. Fourth, although participants with overt inflammatory conditions were excluded and CRP levels were within the normal range, the presence of subclinical inflammation cannot be completely excluded. The multivariable regression model explained 23.4% of the variability in plasma omentin-1 levels, indicating that additional biological and clinical factors not included in the present model may contribute to circulating omentin-1 concentrations. Factors such as body composition, adiposity-related parameters, dietary status, physical activity, and other metabolic or inflammatory mediators may influence omentin-1 levels. However, these variables were not available in the current dataset. In addition, socioeconomic status and dietary iron intake were not specifically assessed. Fifth, no functional mechanistic studies were performed to directly confirm the biological role of omentin-1 in iron metabolism. Finally, the absence of assay standardization for circulating omentin-1 remains an important limitation, as differences in ELISA kits, calibration procedures, and analytical performance may contribute to variability in reported concentrations across studies [26]. Despite these limitations, our study has several strengths, including well-defined diagnostic criteria for IDA and β-thalassemia trait, standardized laboratory measurements, and comprehensive statistical analyses. Importantly, this is among the few studies to simultaneously evaluate omentin-1 across two distinct forms of hypochromic microcytic anaemia and healthy controls.

## 5. Conclusions

The findings of this study suggest that omentin-1 is significantly associated with iron status and erythroid indices and may reflect underlying disturbances in iron metabolism. Omentin-1 showed good discrimination between patients with hypochromic microcytic anaemia and healthy controls, suggesting that it may provide complementary information during the evaluation of microcytic anaemia when interpreted together with established haematological and iron-related parameters. However, its ability to differentiate IDA from β-thalassemia trait was limited, indicating that it cannot replace conventional diagnostic markers. Further large-scale, multicentre, longitudinal studies are needed to clarify the mechanistic role of omentin-1 in iron homeostasis and to determine whether its incorporation into current diagnostic approaches provides clinically meaningful added value beyond existing biomarkers including red blood cell indices, the Mentzer index, iron studies (ferritin and transferrin saturation), and HbA2 quantification. Future studies should also investigate changes in circulating omentin-1 levels following iron supplementation and examine its relationship with erythropoietic regulators, particularly erythropoietin.

## Figures and Tables

**Table 1:** Clinical and Biochemical Characteristics of the Study Population.

Parameter ^1,2^	*n* = 90, Median (Q1–Q3)	Minimum–Maximum
Age (years)	27.00 (22.75–38.00)	18.00–69.00
Omentin-1 (ng/mL)	43.70 (32.45–96.75)	2.00–376.80
HbA1c (%)	5.30 (5.10–5.50)	4.40–6.30
HbF (%)	0.50 (0.30–0.93)	0.20–2.90
HbA0 (%)	86.00 (84.15–86.70)	81.10–88.30
HbA2 (%)	2.80 (2.70–4.80)	2.10–6.00
RBC (×10^12^/L)	5.31 (4.79–5.98)	3.88–7.20
Hb (g/dL)	12.58 (11.55–13.97)	7.50–16.99
MCV (fL)	80.45 (68.18–88.19)	58.60–97.33
MCH (pg)	25.42 (20.24–28.84)	17.37–32.53
MCHC (g/dL)	31.02 (29.30–32.49)	26.90–35.40
RDW (%)	13.60 (12.35–14.45)	11.03–22.54
Mentzer Index (%)	16.37 (11.70–18.04)	8.48–23.45
Ferritin (µg/L)	22.00 (11.40–57.28)	2.90–196.60
Iron (µg/dL)	71.50 (43.00–111.63)	10.00–228.00
TIBC (µg/dL)	237.50 (194.50–333.50)	123.00–524.80
Glucose (mg/dL)	87.00 (81.75–96.25)	64.00–137.00
TC (mg/dL)	151.50 (132.75–179.25)	98.00–294.00
TG (mg/dL)	87.00 (68.75–124.50)	17.00–440.00
HDL-C (mg/dL)	45.05 (37.65–54.15)	23.60–111.20
LDL-C (mg/dL)	89.05 (71.50–104.75)	36.00–179.10
CRP (mg/L) ^3^	<5	<5

^1^ RBC, red blood cell count; Hb, haemoglobin; MCV, mean corpuscular volume; MCH, mean corpuscular haemoglobin; MCHC, mean corpuscular haemoglobin concentration; RDW, Mentzer Index, MCV/RBC; red cell distribution width; TIBC, total iron-binding capacity; CRP, C-reactive protein; TC, total cholesterol; TG, triglyceride; HDL-C, high density-lipoprotein cholesterol; LDL-C, low-density lipoprotein cholesterol. ^3^ CRP values are presented as below the clinical cut-off value (<5 mg/L) because all participants had CRP levels below this threshold. ^2^ Reference ranges and diagnostic cut-off values used in the study were as follows: HbA1c, 4.0–6.4%; HbF, <1.2%; HbA0, >95%; HbA2, <3.5% (β-thalassemia trait: ≥3.5%); RBC, 4.35–5.65 ×10^12^/L in men and 3.92–5.13 ×10^12^/L in women; haemoglobin, ≥13 g/dL in adult men and ≥12 g/dL in non-pregnant adult women; MCV, 80–100 fL; MCH, 27–34 pg; MCHC, 32–36 g/dL; RDW, 11.5–14.5%; Mentzer Index, <13 suggestive of β-thalassemia trait and >13 suggestive of iron-deficiency anaemia; ferritin, 30–322 µg/L in men and 15–291 µg/L in women; serum iron, 65–175 µg/dL in adult men and 50–170 µg/dL in adult women; TIBC, 240–450 µg/dL; glucose, 70–100 mg/dL; total cholesterol, <200 mg/dL; triglycerides, <150 mg/dL; HDL-C, >40 mg/dL in adult men and >50 mg/dL in adult women; LDL-C, <130 mg/dL; and CRP, <5 mg/L.

**Table 2:** Comparison of Biochemical Parameters Between Male and Female Participants.

Parameter ^1^	Male (*n* = 46) Median (Q1–Q3)	Female (*n* = 44) Median (Q1–Q3)	*p*-Value	Effect Size
Age (years)	32.50 (25.00–47.25)	24.50 (21.00–31.50)	<0.001	r = 0.38
Omentin-1 (ng/mL)	38.25 (27.95–60.02)	58.40 (37.38–192.22)	0.014	r = 0.26
HbA1c (%)	5.40 (5.10–5.60)	5.30 (5.13–5.40)	NS	NS
HbF (%)	0.50 (0.30–1.00)	0.50 (0.40–0.90)	NS	NS
HbA0 (%)	85.80 (83.15–87.03)	86.10 (84.70–86.70)	NS	NS
HbA2 (%)	2.90 (2.70–4.83)	2.80 (2.60–4.70)	NS	NS
RBC (×10^12^/L)	5.54 (5.10–6.20)	4.89 (4.43–5.73)	<0.001	r = 0.38
Hb (g/dL)	13.25 (12.49–15.79)	11.87 (11.43–12.79)	<0.001	r = 0.42
MCV (fL)	81.57 (68.34–89.16)	80.45 (68.12–87.39)	NS	NS
MCH (pg)	23.26 (19.87–29.41)	25.59 (20.85–28.32)	NS	NS
MCHC (g/dL)	31.01 (29.18–32.68)	31.02 (29.40–32.42)	NS	NS
RDW (%)	13.49 (12.28–14.77)	13.72 (12.48–14.45)	NS	NS
Mentzer Index (%)	15.36 (11.57–17.31)	16.94 (12.34–19.87)	0.014	r = 0.26
Ferritin (µg/L)	53.15 (14.98–79.82)	16.00 (6.88–28.98)	<0.001	r = 0.41
Iron (µg/dL)	88.00 (53.25–131.75)	56.50 (40.25–93.80)	0.016	r = 0.25
TIBC (µg/dL)	208.50 (173.00–278.25)	267.50 (220.00–375.50)	0.003	r = 0.32
Glucose (mg/dL)	91.00 (85.00–100.25)	84.5 (78.25–92.00)	0.001	r = 0.32
TC (mg/dL)	148.50 (130.00–182.00)	153.50 (135.00–177.75)	NS	NS
TG (mg/dL)	92.50 (73.50–137.00)	79.50 (59.00–91.50)	0.028	r = 0.23
HDL-C (mg/dL)	38.90 (35.75–45.30)	51.35 (45.48–59.65)	<0.001	r = 0.54
LDL-C (mg/dL)	91.45 (72.62–113.17)	87.10 (64.30–101.25)	NS	NS
CRP (mg/L) ^2^	<5	<5	NS	NS

^1^ RBC, red blood cell count; Hb, haemoglobin; MCV, mean corpuscular volume; MCH, mean corpuscular haemoglobin; MCHC, mean corpuscular haemoglobin concentration; RDW, Mentzer Index, MCV/RBC; red cell distribution width; TIBC, total iron-binding capacity; CRP, C-reactive protein; TC, total cholesterol; TG, triglyceride; HDL-C, high-density lipoprotein cholesterol; LDL-C, low-density lipoprotein cholesterol, NS; non-significant. The *p*-values were considered statistically significant at *p* < 0.05. ^2^ CRP values are presented as below the clinical cut-off value (<5 mg/L) because all participants had CRP levels below this threshold.

**Table 3:** Clinical and Biochemical Comparisons of Hypochromic Microcytic Anaemia and Healthy Controls.

Parameter ^1^	Hypochromic Microcytic Anaemia (*n* = 60) Median (Q1–Q3)	Healthy Controls (*n* = 30) Median (Q1–Q3)	*p*- Value	Effect Size
Age (years)	28.00 (23.00–41.50)	27.00 (21.75–31.25)	NS	NS
Omentin-1 (ng/mL)	36.40 (26.68–58.40)	147.65 (47.60–267.23)	<0.001	r = 0.59
HbA1c (%)	5.38 (5.20–5.57)	5.30 (4.98–5.50)	NS	NS
HbF (%)	0.70 (0.40–1.10)	0.40 (0.30–0.40)	<0.001	r = 0.42
HbA0 (%)	85.25 (83.05–86.56)	86.55 (86.10–87.30)	<0.001	r = 0.48
HbA2 (%)	3.65 (2.63–5.00)	2.80 (2.70–2.90)	0.018	r = 0.25
RBC (×10^12^/L)	5.58 (4.87–6.17)	5.04 (4.49–5.46)	0.004	r = 0.38
Hb (g/dL)	11.79 (11.32–12.90)	15.29 (12.98–16.00)	<0.001	r = 0.41
MCV (fL)	71.69 (65.54–80.67)	89.75 (87.38–93.15)	<0.001	r = 0.74
MCH (pg)	21.17 (19.31–25.51)	29.76 (28.32–30.50)	<0.001	r = 0.77
MCHC (g/dL)	29.99 (28.90–31.84)	32.80 (31.95–33.68)	<0.001	r = 0.02
RDW (%)	14.15 (13.46–15.12)	12.23 (11.81–12.71)	<0.001	r = 0.66
Mentzer Index (%)	12.59 (10.53–16.89)	17.91 (17.19–19.35)	<0.001	r = 0.54
Ferritin (µg/L)	14.95 (7.88–45.68)	41.35 (18.35–83.25)	0.014	r = 0.38
Iron (µg/dL)	57.00 (41.25–97.25)	92.60 (60.25–137.75)	0.003	r = 0.31
TIBC (µg/dL)	247.50 (203.25–362.00)	213.00 (166.75–268.50)	0.004	r = 0.30
Glucose (mg/dL)	89.60 (82.25–99.75)	86.00 (78.75–90.75)	0.002	r = 0.32
TC (mg/dL)	146.50 (130.00–160.00)	172.00 (153.00–197.00)	0.002	r = 0.33
TG (mg/dL)	87.50 (63.25–124.50)	79.50 (73.50–126.25)	NS	NS
HDL-C (mg/dL)	43.70 (37.25–53.07)	47.20 (37.65–58.28)	NS	NS
LDL-C (mg/dL)	84.48 (64.68–93.88)	99.30 (88.25–125.95)	0.003	r = 0.31
CRP (mg/L)	2.00 (2.00–2.00)	2.00 (2.00–2.00)	NS	NS

^1^ RBC, red blood cell count; Hb, haemoglobin; MCV, mean corpuscular volume; MCH, mean corpuscular haemoglobin; MCHC, mean corpuscular haemoglobin concentration; Mentzer Index, MCV/RBC; RDW, red cell distribution width; TIBC, total iron-binding capacity; CRP, C-reactive protein; TC, total cholesterol; TG, triglyceride; HDL-C, high-density lipoprotein cholesterol; LDL-C, low-density lipoprotein cholesterol, NS; non-significant. The *p*-values were considered statistically significant at *p* < 0.05.

**Table 4:** Clinical and Biochemical Comparisons of β-thalassemia trait, Iron-Deficiency Anaemia and Healthy Controls.

Parameter ^1^	β-thalassemia trait Median (Q1–Q3)	Iron-Deficiency Anaemia Median (Q1–Q3)	Healthy Controls Median (Q1–Q3)	*p*-Value ^2^	Effect Size
Age (years)	28.00 (24.00–38.00)	32.00 (22.50–49.00)	27.00 (21.75–31.25)	NS	NS
Omentin-1(ng/mL)	43.00 (26.50–79.20)	32.80 (26.74–39.15) ^b^	147.65 (47.60–267.23) ^c^	<0.001	ε^2^ = 0.38
HbA1c (%)	5.30 (5.00–5.48)	5.40 (5.30–5.61)	5.30 (4.98–5.50)	NS	NS
HbF (%)	1.10 (0.70–1.60) ^a^	0.40 (0.30–0.65)	0.40 (0.30–0.40) ^c^	<0.001	ε^2^ = 0.51
HbA0 (%)	83.20 (82.30–84.20) ^a^	86.60 (85.80–86.95)	86.55 (86.10–87.30) ^c^	<0.001	ε^2^ = 0.64
HbA2 (%)	5.00 (4.80–5.60) ^a^	2.60 (2.50–2.80)	2.80 (2.70–2.90) ^c^	<0.001	ε^2^ = 0.72
RBC (×10^12^/L)	6.16 (5.74–6.52) ^a^	4.88 (4.64–5.48)	5.04 (4.49–5.46) ^c^	<0.001	ε^2^ = 0.46
Hb (g/dL)	12.05 (11.30–13.25)	11.65 (11.37–12.54) ^b^	15.29 (12.98–16.00) ^c^	<0.001	ε^2^ = 0.45
MCV (fL)	65.58 (63.70–71.15) ^a^	80.00 (74.09–85.24) ^b^	89.75 (87.38–93.15) ^c^	<0.001	ε^2^ = 0.71
MCH (pg)	19.37 (18.60–21.29) ^a^	25.25 (21.25–26.74) ^b^	29.76 (28.32–30.50) ^c^	<0.001	ε^2^ = 0.71
MCHC (g/dL)	29.19 (28.69–30.30)	30.89 (29.99–31.99) ^b^	32.80 (31.95–33.68) ^c^	<0.001	ε^2^ = 0.44
RDW (%)	13.81 (13.30–14.70)	14.45 (13.78–15.85) ^b^	12.23 (11.81–12.71)	<0.001	ε^2^ = 0.46
Mentzer Index (%)	10.56 (9.72–11.87) ^a^	16.64 (13.52–18.07) ^b^	17.91 (17.19–19.35)	<0.001	ε^2^ = 0.59
Ferritin (µg/L)	43.80 (20.70–76.69) ^a^	7.70 (3.70–11.10) ^b^	41.35 (18.35–83.25)	<0.001	ε^2^ = 0.56
Iron (µg/dL)	95.00 (66.00–120.00) ^a^	42.00 (28.00–53.00) ^b^	92.60 (60.25–137.75)	<0.001	ε^2^ = 0.42
TIBC (µg/dL)	207.00 (181.00–242.00) ^a^	362.00 (274.00–447.50) ^b^	213.00 (166.75–268.50)	<0.001	ε^2^ = 0.40
Glucose (mg/dL)	87.00 (82.00–98.00)	89.60 (82.50–106.50)	86.00 (78.75–90.75)	NS	NS
TC (mg/dL)	146.00 (130.00–167.00)	149.00 (130.00–159.00) ^b^	172.00 (153.00–197.00) ^c^	0.007	ε^2^ = 0.09
TG (mg/dL)	83.00 (70.00–95.00)	90.00 (52.00–135.50)	79.50 (73.50–126.25)	NS	NS
HDL-C (mg/dL)	45.90 (38.00–53.30)	40.40 (36.50–53.45)	47.20 (37.65–58.28)	NS	NS
LDL-C (mg/dL)	89.00 (64.10–98.40)	80.00 (64.70–92.50)	99.30 (88.25–125.95) ^c^	NS	NS
CRP (mg/L)	2.00 (2.00–2.00)	2.00 (2.00–2.15)	2.00 (2.00–2.00)	NS	NS

^1^ RBC, red blood cell count; Hb, haemoglobin; MCV, mean corpuscular volume; MCH, mean corpuscular haemoglobin; MCHC, mean corpuscular haemoglobin concentration; Mentzer Index, MCV/RBC; RDW, red cell distribution width; TIBC, total iron-binding capacity; CRP, C-reactive protein; TC, total cholesterol; TG, triglyceride; HDL-C, high-density lipoprotein cholesterol; LDL-C, low-density lipoprotein cholesterol, NS; not significant. ^2^ In post hoc pairwise analyses, a indicates a significant difference between the β-thalassemia trait and iron-deficiency anaemia groups; b indicates a significant difference between the iron-deficiency anaemia and healthy control groups; and c indicates a significant difference between the β-thalassemia trait and healthy control groups. The *p*-values for a, b, and c were adjusted using the Bonferroni correction for multiple comparisons and were considered statistically significant at *p* < 0.05.

**Table 5:** Correlations between plasma omentin-1 levels and clinical, haematological, iron-related, and biochemical parameters.

Parameter ^1^	Spearman r	*p*-Value
Age (years)	−0.296	0.005
HbA1c (%)	−0.229	0.030
HbA0 (%)	0.211	0.046
RBC (×10^12^/L)	−0.212	0.045
Hb (g/dL)	0.236	0.025
MCV (fL)	0.382	<0.001
MCH (pg)	0.350	0.001
MCHC (g/dL)	0.284	0.007
RDW (%)	−0.520	<0.001
Mentzer Index	0.322	0.002
Ferritin (µg/L)	0.255	0.015
Iron (µg/dL)	0.286	0.006
TIBC (µg/dL)	−0.244	0.020
Glucose (mg/dL)	−0.296	0.005
TC (mg/dL)	0.310	0.003
HDL-C (mg/dL)	0.255	0.015
LDL-C (mg/dL)	0.280	0.007

^1^ RBC, red blood cell count; Hb, haemoglobin; MCV, mean corpuscular volume; MCH, mean corpuscular haemoglobin; MCHC, mean corpuscular haemoglobin concentration; Mentzer Index, MCV/RBC; RDW, red cell distribution width; TIBC, total iron-binding capacity; TC, total cholesterol; HDL-C, high-density lipoprotein cholesterol; LDL-C, low-density lipoprotein cholesterol. The *p*-values were considered statistically significant at *p* < 0.05.

## Data Availability

The raw data supporting the conclusions of this article will be made available by the corresponding author on request. The data are not publicly available due to ethical and privacy restrictions related to the protection of participant confidentiality.

## Abbreviations

The following abbreviations are used in this manuscript:

AUC	Area under the curve
CRP	C-reactive protein
ELİSA	Enzyme-linked immunosorbent assay
Hb	Haemoglobin
HbA1c	Glycated haemoglobin
Hct	Haematocrit
HDL-C	High-density lipoprotein cholesterol
IDA	Iron-deficiency anaemia
LDL-C	Low-density lipoprotein cholesterol
MCV	Mean corpuscular haemoglobin
MCHC	Mean corpuscular haemoglobin concentration
RBC	Red blood cell
RDW	Red cell distribution width
ROC	Receiver operating characteristic
TG	Triglyceride
